# Estimation of the Seasonal Inhaled Deposited Dose of Particulate Matter in the Respiratory System of Urban Individuals Living in an Eastern Mediterranean City

**DOI:** 10.3390/ijerph19074303

**Published:** 2022-04-03

**Authors:** Tareq Hussein, Asal Al-Abdallat, Shatha Suleiman Ali Saleh, Marwan Al-Kloub

**Affiliations:** 1 Department of Physics, School of Science, The University of Jordan, Amman 11942, Jordan; asl9170208@ju.edu.jo (A.A.-A.); mkloob@gmail.com (M.A.-K.); 2 Institute for Atmospheric and Earth System Research (INAR/Physics), University of Helsinki, FI-00014 Helsinki, Finland; 3 Department of Basic Sciences, National University College of Technology, Amman 11191, Jordan; shasaleh@nuct.edu.jo

**Keywords:** urban aerosols, human activities, regional inhaled dose, particle size distribution, PM_2.5_

## Abstract

In this study, we present an estimation for the inhaled deposited dose rate in adult males and females during common exposure scenarios to urban background aerosols in an Eastern Mediterranean city (Amman, Jordan) based on a one-year database of measured particle number size distribution. The dose rates show seasonal variations reflecting the physical characteristics (i.e., modal structure) of the particle number size distribution. An additional factor was the varying deposition fraction (*DF*) for different regions and different human activities (exercising versus resting). The total dose rate was 3 × 10^9^–65 × 10^9^ particles/h (PM_2.5_ and PM_10_ doses 1–22 µg/h and 9–210 µg/h; respectively) depending on the gender, activity, and season. Based on the particle number metrics, the inhaled deposited dose in the head, Tracheobronchial, and alveolar were 7–16%, 16–28%, and 56–76%; respectively. Based on the PM_2.5_ metric, the corresponding dose rate was 9–41%,13–19%; and 46–72% respectively. As for the PM_10_ metric, they were 25–75%, 7–35%, and 15–55%; respectively.

## 1. Introduction

Urban aerosols have gained increased attention over the past decades because they have clear health effects related to their exposure [1,2]. Health studies and air quality regulations have been based on the mass concentration of particulate matter (PM); however, recent studies have increased the awareness about the importance of the number concentration as a complementary metric needed to understand the health effects and exposure. This has been evident from the fact that submicron particles penetrate deep into the respiratory system reaching the alveolar region, where the blood can carry particulate matter and circulate them in different organs causing serious health effects in the human body [3]. Besides their health effects, ultrafine particles (UFP, diameter < 0.1 µm) can act as cloud condensation nuclei (CCN) affecting the climate [4,5].

Ultrafine particles (UFP, diameter < 0.1 µm) do not have a significant fraction in the total mass concentration but they have a significant fraction in the number concentration in urban areas. Besides that, UFP emanate from a complex variety of sources (primary and secondary) that are directly linked to anthropogenic activities. Primary particles are directly emitted into the atmosphere. Secondary particles are produced in the atmosphere via gas-to-particle transformation, which has been known as new particle formation (NPF) observed in various environments and contributing to a major fraction of the total particle number budget [6,7,8]. The complexity of urban aerosols comes from the fact that different sources may entangle them in the same particle size range that becomes difficult to distinguish them without advanced chemical-physical characterization and analysis [9,10]. This leads to further difficult task to understand the toxicity and health effects of urban aerosols.

Recently, several studies focused on the urban particle number size distribution in some cities in the East Mediterranean region [11,12,13,14,15,16,17]. However, exposure assessment and estimation of the inhaled deposited dose have never been studied in this region extensively [18,19]. Therefore, the aim of this study was to investigate the inhaled deposited dose rate in adult males/females living in an urban background area in Amman, Jordan. The calculation of the inhaled dose was made by utilizing our previous one-year extensive measurement campaign for the particle number size distribution (0.01–10 µm) at an urban background location in Amman [20]. We considered common human activities (exercising and resting) to reflect realistic conditions of exposure.

## 2. Materials and Methods

### 2.1. Aerosol Database and Measurement Site Description

The aerosol database was adopted from our previously measured aerosol particle number size distributions in the urban background atmosphere of Amman, Jordan. This database was analyzed extensively in our previously published papers by Hussein et al. [20]. The details of the measurement site, experimental setup, and data handling are in the Supplementary Material (Sections S1–S3).

The measurement location was at the Environmental and Atmospheric Research Laboratory (EARL), which was located on the third floor of the Department of Physics, University of Jordan. The University of Jordan campus was located in an urban environment in the north part of Amman, Jordan (Supplementary Material Figure S1). The selection of this site was for the purpose of being representative of an urban background atmosphere and reflecting an exposure scenario not affected by close/nearby strong urban sources of air pollution. The measurement location is a central point at the campus of the University of Jordan, which complies with the fact that is an urban background location not directly influenced by traffic emissions or industrial activities. In other words, the sampled aerosols represented urban background conditions transported from the city in different directions (i.e., down wind). As such, there was no need to correct for the elevation of the measurement from the ground. The sampling was seen as suitable for the study instead of having the measurements nearby or close to the sources.

The database included the particle number size distributions (particle diameter 0.01–10 µm) measured during 1 August 2016–31 July 2017. The particle number size distributions were measured with a scanning mobility particle sizer (NanoScan SMPS 3910, TSI, Shoreview, MN, USA), which has an electrical equivalent mobility diameter range of 0.01–0.42 µm with 13 channels under dry conditions and 1-min scan time, and an optical particle sizer (OPS 3330, TSI, Shoreview, MN, USA), which has an optical diameter range 0.3–10 µm with 13 channels at dry conditions and 5 min scan time. Each instrument had its own aerosol sampling inlet (~1-m-long and 8 mm inner diameter) which was led through the wall to sample the outdoor aerosols. Each inlet consisted of short Tygon tubes (4 mm inner diameter) connected to a diffusion drier (TSI model 3062-NC). The aerosol transport efficiency through the aerosol inlet assembly was determined experimentally (Figure S2). The penetration efficiency was ~47% for 10 nm, ~93% for 0.3 µm, and ~40% for 10 µm particles.

In order to construct a wide range (0.01–10 µm) of the measured particle number size distribution, we performed the following steps:

1. Calculated the 5-min average of the SMPS data;
2. Omitted the last two channels in the SMPS (i.e., remaining diameter range was 0.01–0.25 µm);
3. Omitted the first channel in the OPS (i.e., the remaining diameter range was 0.32–10 µm);
4. Merged the two distributions.

### 2.2. Regional Inhaled Deposited Dose

According to the ICRP and MPPD models [21,22], the respiratory tract is divided into three regions: head/throat, tracheobronchial (TB), and pulmonary/alveolar (P/Alv). Following our previous methodology [18,19,23], the regional inhaled deposited dose can be calculated for a specific particle diameter range (*D*_*p*1_–*D*_*p*2_) during a one-hour exposure period, which is known as the dose rate:

(1) $$\mathrm{Dose}\;\mathrm{Rate}=\int_{D_{P}1}^{D_{P}2}V_{E}\cdot DF\left(D_{P}\right)\cdot n_{N}^{0}\left(D_{P}\right)\cdot f\mathrm{dlog}\left(D_{P}\right),$$

where *V_E_* is the minute ventilation (volume of air breathed as reported by Holmes [24], Table S1), *DF*(*D_p_*) is the aerosol deposition fraction in a particular region of the respiratory tract (Figure S1, Löndahl et al. [25]), *n_N_*^0^(*D_p_*) (particles/cm^3^) is the particle number size distribution (i.e., d*N*/dlog(*D_p_*)), and *f* is a metric conversion for the aerosol concentration (i.e., it is 1 for particle number and for particle mass = *ρ_p_D_p_*^3^π/6, where *ρ*_p_ is the particle effective density). Note that the inhaled deposition fraction (*DF(D_p_)*) and the particle number size distribution ($n_{N}^{0}\left(D_{P}\right)$) are functions of particle diameter (*D_p_*).

The details about the calculation of the regional inhaled deposited dose rate are described in the Supplementary Material (Section S4). The results herein are presented based on the mean particle number size distribution. As a measure of the uncertainty of the calculation, we shall discuss the results with respect to the quartiles of the aerosol concentrations.

### 2.3. Exposure Scenarios

The regional inhaled deposited dose rates were calculated for adult male and female occupants during different activity levels:

1. Resting, such as sitting and standing.
2. Exercising, such as walking, running, and yard working.

The combination of subjects (male/female), activities (resting/exercising), and exposure levels (different time of the year, month, week, and day) reflect common exposure scenarios in the urban background atmosphere of Amman, which can be a good example for an Eastern Mediterranean city. The required respiratory tract parameters (i.e., *V_E_* and *DF*) are described in detail in Figure S3 and Table S1. As for the exposure parameters, these are taken from the aerosol database for the particle number size distribution (*n_N_*^0^(*D_p_*)).

## 3. Results and Discussion

### 3.1. Summary of the Particulate Matter Concentrations

The ultrafine particle number concentration (PN_UFP_, diameter < 0.1 µm) fraction was about 93% of PN_Sub_ concentration. The accumulation mode particle number concentration (PN_ACCU_, diameter 0.1–1 µm) was about 7% of PN_Sub_. The submicron particle number concentrations (PN_Sub_) and its particle fractions (PN_UFP_ and PN_ACCU_) showed clear temporal variations (Figures S4–S8 and Table S2):

- Seasonal: submicron particle number concentration (PN_Sub_) was higher during the winter (December–February, range 3.3 × 10^4^–3.7 × 10^4^ cm^−3^) than during the summer (June–September, 1.2 × 10^4^–1.6 × 10^4^ cm^−3^). These numbers were according to the daily means.
- Weekly: The PN_Sub_ concentrations during the weekends (Friday–Saturday, peak value < 2.7 × 10^4^ cm^−3^) were lower than on workdays (Sunday–Thursday peak value > 3.3 × 10^4^ cm). These numbers were according to the hourly means.
- Diurnal pattern: (1) workdays were characterized by two peaks (morning ~4.1 × 10^4^ cm^−3^ and afternoon ~2.9 × 10^4^ cm^−3^) related to traffic rush hours, (2) weekends (Friday–Saturday) were characterized by a wide peak during the day, and (3) the lowest concentrations were below 1.5 × 10^4^ cm^−3^ before 6 a.m. regardless of the weekday.

The coarse mode particle number (PN_Coarse_) concentration had a different seasonal pattern than that of PN_Sub_. PN_Coarse_ was the highest during the autumn and spring. This seasonal pattern is closely linked to the sand and dust storms (SDS) in the spring season and local dust resuspension in the autumn. The daily average PN_Coarse_ often exceeded 2 cm^−3^ (as high as 14.5 cm^−3^) during SDSs and the hourly average reached values as high as 46 cm^−3^.

As shown in Figure S9, the particle number size distribution also showed clear variation in its modal structure with respect to the time of the year, as well as the time of the day. For example, the UFP modes had higher concentrations during the winter (December–February) than in the summer (June–August). During the traffic rush hours (morning and afternoon) the UFP particle modes also had higher concentrations than during the early morning (before 6:00).

### 3.2. Inhaled Deposited Dose

#### 3.2.1. Total Inhaled Deposited Dose Rate

**Figure 1:**
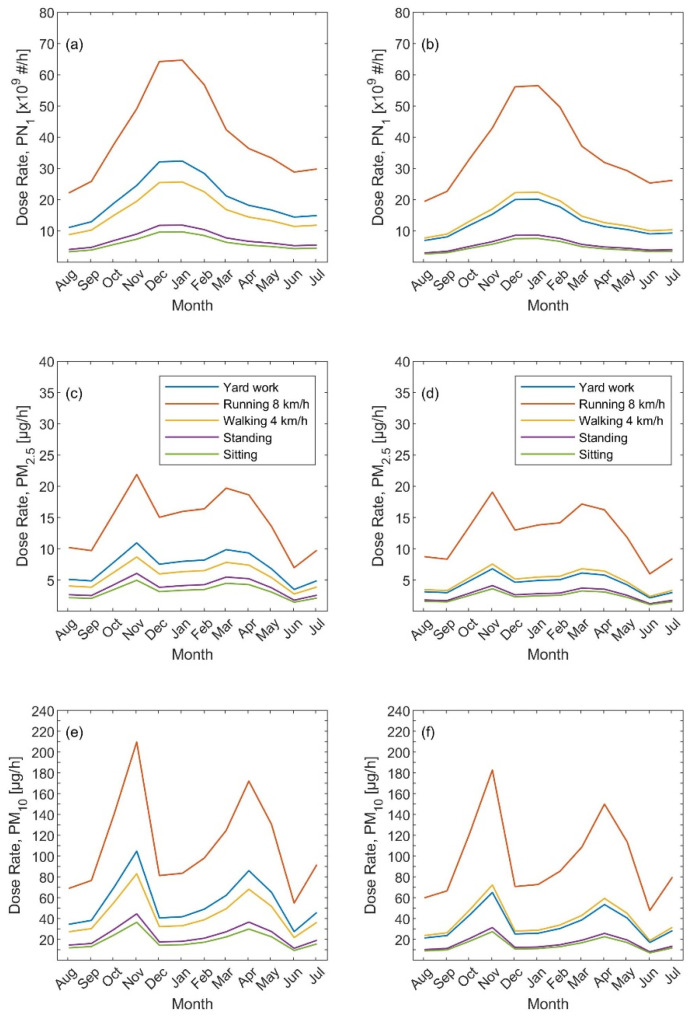
Seasonal variation (monthly averages) of the total inhaled deposited rate with respect to three metrics and five human activities (yard work, running, walking, standing, and sitting): (**a**,**b**) particle number, (**c**,**d**) PM_2.5_, and (**e**,**f**) PM_10_. The left panel is the inhaled dose rate calculated for an adult male and the right panel for an adult female.

The total inhaled deposited dose rate based on the particle number metrics (i.e., PN_Sub_) showed clear strong seasonal variation with high dose rates during the winter and low dose rates during the summer (Figure 1a,b). For example, the monthly total dose rate was 22 × 10^9^–65 × 10^9^, 11 × 10^9^–32 × 10^9^, 9 × 10^9^–25 × 10^9^, 4 × 10^9^–12 × 10^9^, and 3 × 10^9^–10 × 10^9^ particles/h for an adult male during the following activities: running (8 km/h), yard work, walking (4 km/h), standing, and sitting; respectively. The corresponding numbers were slightly lower for adult females: 19 × 10^9^–57 × 10^9^, 8 × 10^9^–22 × 10^9^, 7 × 10^9^–20 × 10^9^, 3 × 10^9^–9 × 10^9^, and 3 × 10^9^–7 × 10^9^ particles/h; respectively. This seasonal variation was identical to the fine particle number concentration shown in Figure S4 (Supplementary Material Section S5). This result was expected because the total dose rate is proportional to the fine particle number concentration. The higher the minute ventilation (i.e., *V_E_*) was the higher the dose rate; the results reflect the highest dose rates for the running activity, and the lowest dose rate was observed for the resting activities.

Interestingly, the seasonal dose rate showed a different cycle when considering the PM_2.5_ metric (Figure 1c,d). In this case, the dose rate was the highest during the autumn (specifically around October and November) and the spring (specifically around March and April). Similar to the particle number metric, the lowest dose rate was also seen in the summer. Quantitively, the monthly total dose rate in the summer (June–August) was around 7, 3.5, 2.7, 1.7, and 1.4 µg/h for an adult male, and for the same order of the above-mentioned activities. During the winter (December–February), the corresponding dose rate was about 15, 7.6, 6, 4, and 3.3 µg/h. During the spring (March–May), the dose rate was as high as 20, 10, 7.5, 5.4, and 4.4 µg/h and during the autumn (September–November), it was as high as 22, 11, 8.5, 6, and 4.8 µg/h. The corresponding dose rates for an adult female were lower than those for an adult male; the ratios between the genders varied between 66% and 88% depending on the activity and the season. The reason for these differences between females and males can be explained by the differences in the body size (i.e., lungs size) and breathing minute ventilation (i.e., *V_E_*). The female lungs’ size is smaller than those of a male, and consequently, their *V_E_* is lower.

With respect to the PM_10_ metric, the differences in the dose rate were marginal between the summer and winter and the highest dose rates were observed during the autumn followed by the spring (Figure 1e,f). Similar to the PM_2.5_ metric, the corresponding dose rate for an adult female was lower than those for an adult male with the ratio between 61% and 91% depending on the activity and the season. Quantitively, the monthly total dose rate in the summer was around 55, 28, 22, 12, and 9 µg/h for an adult male, and for the same order of the above-mentioned activities. During the winter, the corresponding dose rate was about 81, 40, 32, 17, and 14 µg/h. During the spring, the dose rate was as high as 171, 85, 67, 36, and 29 µg/h and during the autumn was as high as 210, 105, 82, 43, and 35 µg/h.

Based on the seasonal variation of the dose rate with respect to the two metrics (i.e., particle number versus particle mass), it is obvious that there is a contradicting result regarding when to avoid high dose rates received in the respiratory system. For example, according to the particle number dose rates, the highest probability of receiving high dose rates is expected in the winter, namely during December and January. However, when considering the particle mass, the highest dose rate is expected to be received during the autumn (namely October and November) and the spring (namely March and April). The important question comes here: which metric shall we consider? In order to answer that, more investigations shall be made to study the health effects with the respect to different aerosols metrics in addition to the composition and toxicity. For instance, the lung deposition surface area (LDSA) is one of the metrics, but this alone will have a limitation to making constructive conclusions.

**Figure 2:**
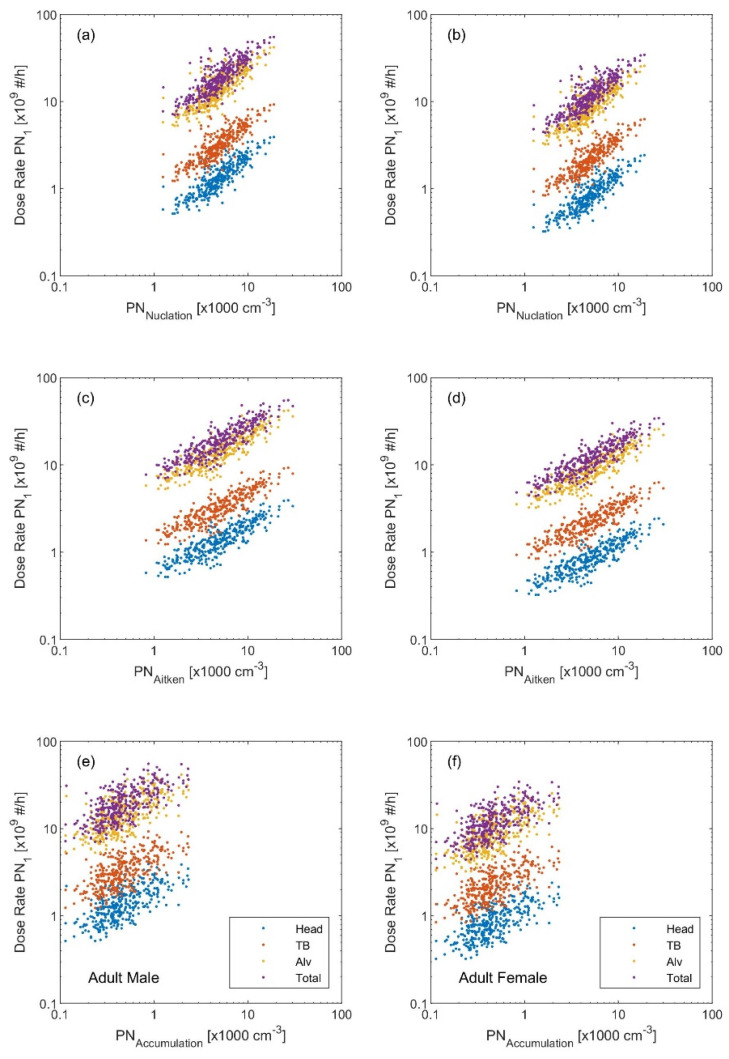
Regression scatter plots between the inhaled deposited dose rate (based on the particle number PN_Sub_ metric) and the particle number concentration of (**a**,**b**) nucleation mode, (**c**,**d**) Aitken mode, and (**e**,**f**) accumulation mode. The left panel is the inhaled dose rate calculated for an adult male and the right panel for an adult female.

**Figure 3:**
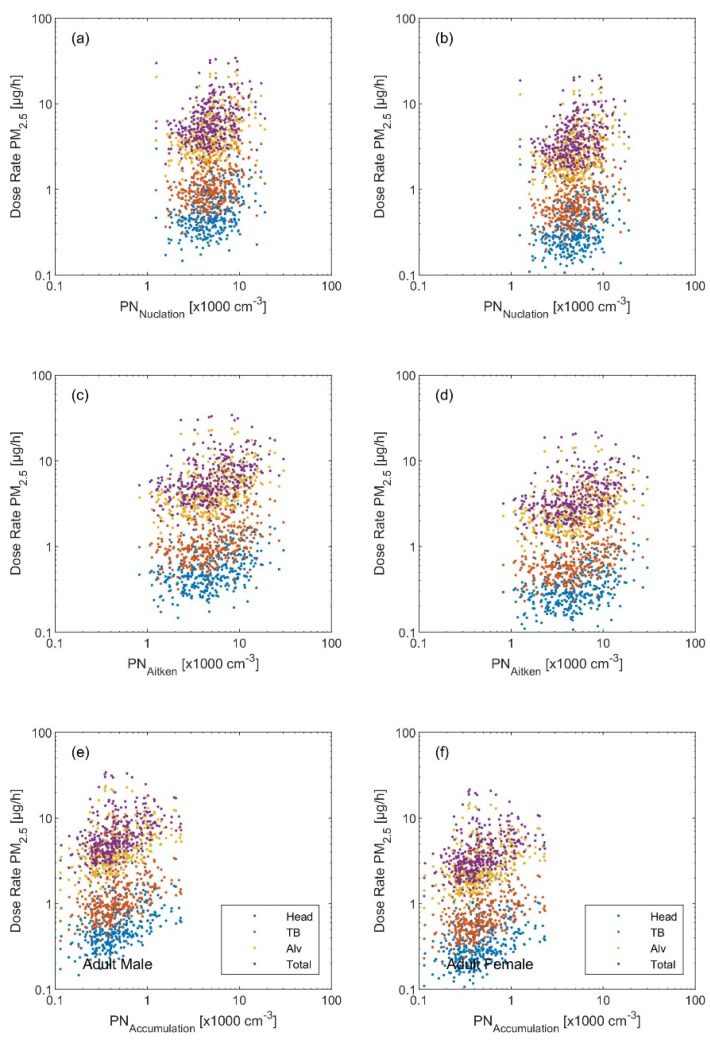
Regression scatter plots between the inhaled deposited dose rate (based on the PM_2.5_ metric) and the particle number concentration of (**a**,**b**) nucleation mode, (**c**,**d**) Aitken mode, and (**e**,**f**) accumulation mode. The left panel is the inhaled dose rate calculated for an adult male and the right panel for an adult female.

**Figure 4:**
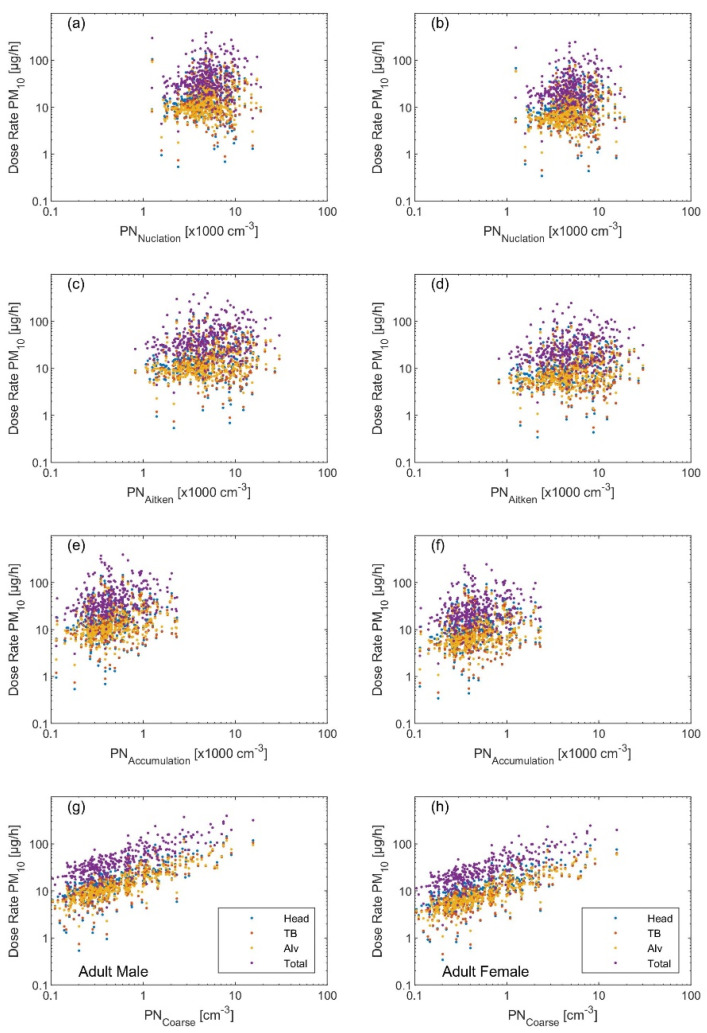
Regression scatter plots between the inhaled deposited dose rate (based on the PM_10_ metric) and the particle number concentration of (**a**,**b**) nucleation mode, (**c**,**d**) Aitken mode, (**e**,**f**) accumulation mode, and (**g**,**h**) coarse mode. The left panel is the inhaled dose rate calculated for an adult male and the right panel for an adult female.

The variation in the dose rates based on the metric calls for an urgent deep investigation to better understand the health effects not only based on the PM_2.5_ but also on the PN_Sub_. This fact is further revealed when looking at the regression plots of each dose rate metric with respect to the modal structure of the particle number size distribution (i.e., simplified as the concentration of each individual mode: nucleation (*D_p_* 10–25 nm), Aitken (*D_p_* 25–100 nm), accumulation (*D_p_* 0.1–1 µm), and coarse (*D_p_* 1–10 µm)) shown in Figure 2, Figure 3 and Figure 4. Quantitively, this is reflected by the *R^2^* and the correlation coefficient (*r*) (Table 1, Table 2 and Table 3). The PN_Sub_ inhaled deposited dose metric had the highest *R^2^* and *r* with the total particle number concentration and the ultrafine particles (diameter < 100 nm). The other two metrics based on the particle mass (i.e., PM_2.5_ and PM_10_) had the highest *R^2^* and *r* with coarse particle size fraction.

#### 3.2.2. Regional Inhaled Deposited Dose Rate

**Figure 5:**
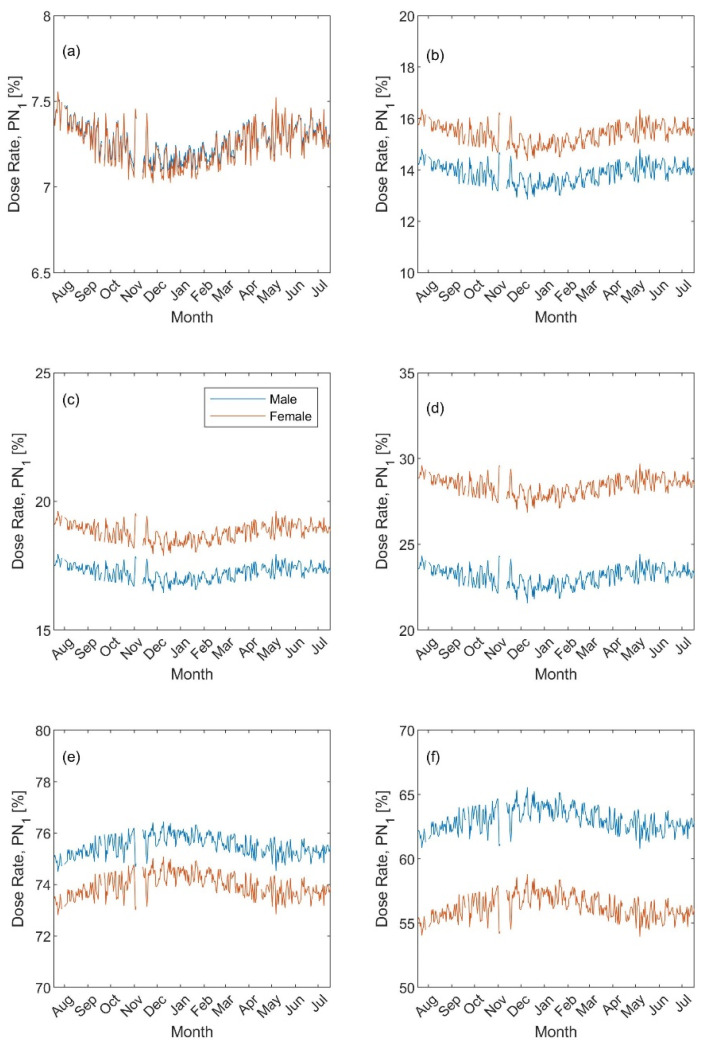
Regional inhaled deposited dose fraction (presented as percentage) based on the particle number PN_Sub_ metric: (**a**,**b**) head, (**c**,**d**) Tracheobronchial, and (**e**,**f**) Alveolar. The left panel for exercising and the right panel for resting.

**Figure 6:**
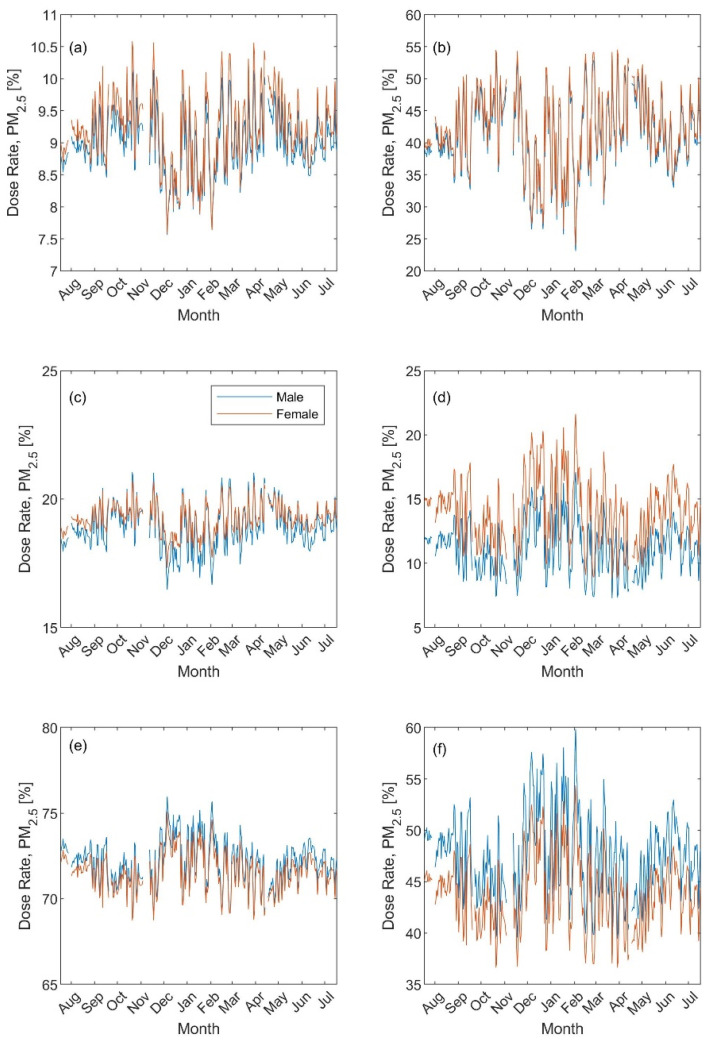
Regional inhaled deposited dose fraction (presented as percentage) based on the PM_2.5_ metric: (**a**,**b**) head, (**c**,**d**) Tracheobronchial, and (**e**,**f**) Alveolar. The left panel for exercising and the right panel for resting.

The regional inhaled deposited dose (Illustrated as the percentage fraction Figure 5 and Figure 6) also showed a clear seasonal variation. Based on the particle number metric, the exercising dose rate (Figure 5a) in the head was rather identical for both males and females and had its minimum value (about 7%) during the winter and its maximum during the summer (about 7.5%). The exercising dose rate in the Tracheobronchial (Figure 5c) also showed a similar seasonal variation but the females had a high fraction (18–20%) than that of the males (16–18%). As for the exercising dose rate received in the Alveolar (Figure 5e), it showed an opposite seasonal variation to those of other regions (i.e., maximum in the winter) and was higher for males (74–76%) than females (73–75%). During resting (Figure 5b,d,f), the differences in the regional dose rates between males and females were more pronounced but followed similar seasonal variations as those for during exercising: around 14%, 23%, 63%, respectively, in the head, Tracheobronchial, and Alveolar, for an adult male versus 16%, 28%, and 56% for an adult female.

**Figure 7:**
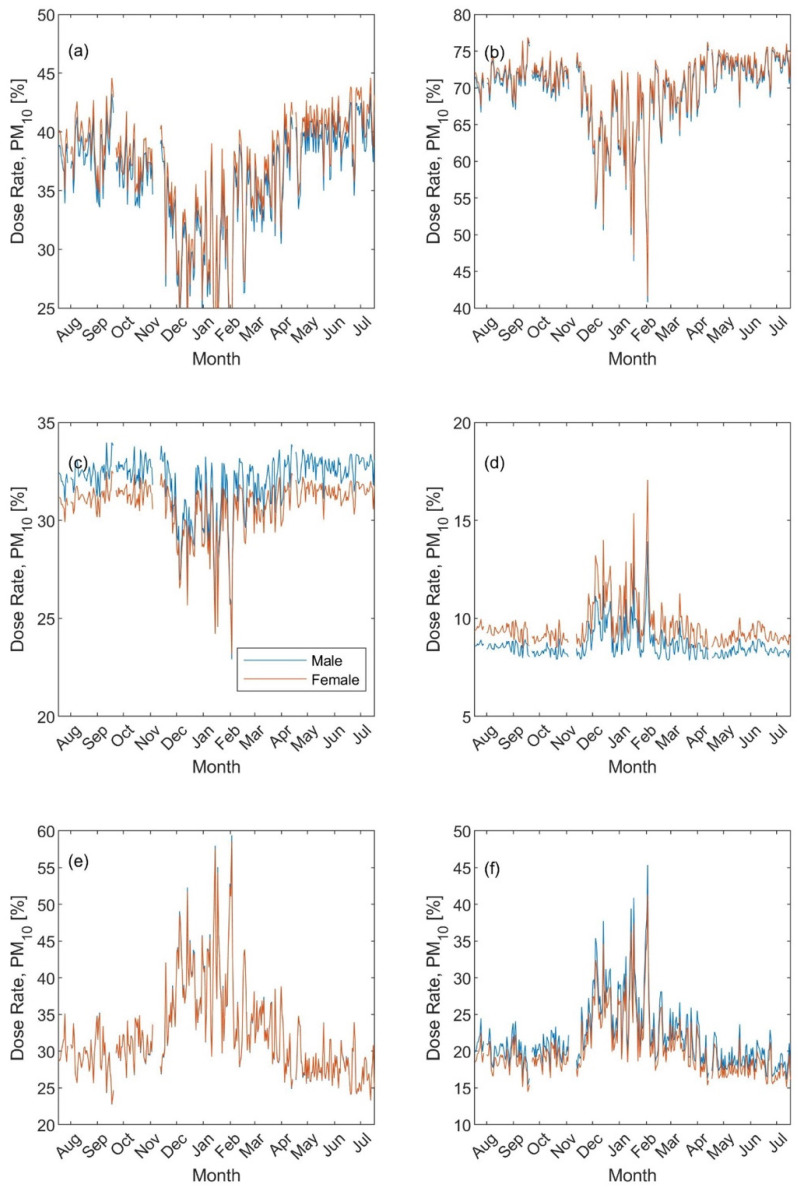
Regional inhaled deposited dose fraction (presented as percentage) based on the PM_10_ metric: (**a**,**b**) head, (**c**,**d**) Tracheobronchial, and (**e**,**f**) Alveolar. The left panel for exercising and the right panel for resting.

Based on the PM metrics, the regional inhaled deposited dose rates were rather similar for males and females. However, the seasonal trend was less pronounced for the PM_2.5_ metric (Figure 6): it was around 9%, 19%; and 72%, respectively, in the head, Tracheobronchial, and Alveolar during exercising versus 41%, 13%, and 46% during resting. As for the PM_10_ metric, the exercising and resting head dose rates were minimum during the winter and varied within 25–45% and 45–75% (Figure 7a,b). In contrast, the exercising and resting Alveolar dose rates were maximum during the winter and varied within 25–55% and 15–45% (Figure 7e,f). The Tracheobronchial dose rate seasonal variation varied between 25–35% and similar to that of the Head during exercising (Figure 7c) and 7–18% and similar to the Alveolar during resting (Figure 7d).

Surprisingly and contrary to what we expected, the regional dose rate seasonal variation for the PM_10_ (illustrated as the percentage fraction) was not similar to that of the coarse mode concentrations (Figure S8). Surprisingly, the PM_2.5_ was the one that showed rather similar seasonal variation to that of the coarse mode concentrations.

In fact, the varying physical characteristics (i.e., modal structure) of the particle number size distribution (Figure S9) through the year (i.e., seasonal variation) was the critical factor in defining the regional inhaled deposited dose rate. This is in addition to the varying deposition fraction (*DF*) for different regions and different human activities (exercising versus resting) (Figure S3).

Recalling the particle number concentrations are dominated by the UFP fraction as discussed in Section 2.1 “Summary about the particulate matter concentrations”, then it can be used to check the accuracy of the calculated dose rate based on the particle number concentrations. For instance, the difference between the monthly mean and the corresponding 25% of the UFP concentrations (and the submicron particle concentrations) was about 40%. Similarly, the difference between the monthly mean and the corresponding 75% of the UFP concentrations was about 30%. This means that the monthly dose rate varies between 30% and 40% based on the particle number concentrations. As for the dose rate calculated in terms of particle mass metrics, the monthly dose rate is expected to vary between 15% and 50%, which reflects the mass concentration variability of the accumulation and coarse modes.

The limitation of this study relies on several factors: (1) the measured particle number concentrations were selected for urban background conditions, (2) the calculations were made by assuming the exposure time as outdoors only, (3) the calculations were made based on seasonal variation, (4) the selected scenarios were limited to adult male/female and four activities, and (5) the conversion from particle number to particle mass concentration has some limitations (such as lack of information about particle density, shape, and chemical composition). The first and second limitations imply that the calculated dose rates here in this study represent a lower estimation for the real dose because urban people are exposed to a variety of environments, which vary from background to polluted areas (i.e., traffic, industry, etc.). Usually, people spend most of their time indoors being exposed to different types and levels of pollutants. The third limitation can be taken care of by recalculating the dose rates by using higher time resolutions (e.g., daily or hourly). The selected scenarios (fourth limitation) can be extended to more realistic human activities such as the typical daily behavior of an urban person. The fifth limitation cannot be taken care of unless detailed aerosol sampling is made aiming at gaining better knowledge about the physical-chemical properties of aerosols. The chemical composition might affect the hygroscopicity of particulate matter; which, in turn, affects the deposition pattern in the respiratory system.

## 4. Conclusions

In this study, we presented an estimation for the inhaled deposited dose rate in adult males and females during common exposure scenarios (i.e., human activities of exercising and resting) to urban background aerosols in an Eastern Mediterranean city. The calculations were based on a one-year database of the measured particle number size distribution in Amman, Jordan. The analysis of the inhaled deposited dose rate included seasonal variation and regional doses in the respiratory system.

A quick glance at the aerosol database indicates that the ultrafine particle number concentration (PN_UFP_, diameter < 0.1 µm) constitutes about 93% submicron aerosols. Aerosols of different particle size fractions showed clear temporal variations: seasonal, weekly, and diurnal reflecting the temporal variation of the anthropogenic activities and influencing factors such as meteorological conditions. The daily mean of the submicron particle number concentration (PN_Sub_) was within the range of 1.2.3 × 10^4^–3.7 × 10^4^ cm^−3^ with high concentrations during the winter. The coarse mode particle number (PN_Coarse_) concentration had a different seasonal pattern with high concentrations during the autumn and spring reflecting sand and dust storms (SDS) in the spring season and local dust resuspension in the autumn. The daily average PN_Coarse_ often exceeded 2 cm^−3^ (as high as 14.5 cm^−3^) during SDS episodes.

The total and regional inhaled deposited dose rates showed seasonal variations. The total dose rate ranged between 3 × 10^9^ and 65 × 10^9^ particles/h (corresponding PM_2.5_ dose 1–22 µg/h and PM_10_ 9–210 µg/h) depending on the gender, activity, and season. Based on the particle number metrics, the inhaled deposited dose in the head, Tracheobronchial, and alveolar were 7–16%, 16–28%, and 56–76%; respectively. Based on the PM_2.5_ metric, the dose rate was 9–41%, 13–19%; and 46–72% respectively in the head, Tracheobronchial, and alveolar. As for the PM_10_ metric, the dose rates were 25–75%, 7–35%, and 15–55% for the head, Tracheobronchial, and alveolar; respectively.

In general, the varying physical characteristics (i.e., modal structure) of the particle number size distribution through the year (i.e., seasonal variation) was the critical factor in defining the regional inhaled deposited dose rate in addition to the varying deposition fraction (*DF*) for different regions and different human activities (exercising versus resting).

## Figures and Tables

**Table 1:** Coefficient of determination (*R^2^*) and correlation coefficient (*r*) calculated for the regional inhaled deposited dose (PN_Sub_) with respect to the size/fractionated particle number concentrations: nucleation mode (NUCL, 10–25 nm), Aitken mode (AITK, 25–100 nm), accumulation mode (ACCU, 0.1–1 µm), coarse mode (COAR, 1–10s µm), and the total diameter range (Total, 10 nm–10 µm).

	Activity	Region	Male					Female				
			NUCL	AITK	ACCU	COAR	Total	NUCL	AITK	ACCU	COAR	Total
***R* ^2^**	**Yard Work**	Total	**0.80**	**0.96**	0.31	0.02	0.99	**0.81**	**0.95**	0.30	0.02	0.99
		Head	**0.81**	**0.95**	0.30	0.02	0.99	**0.82**	**0.95**	0.30	0.02	0.98
		Tracheobronchial	**0.82**	**0.94**	0.30	0.02	0.98	**0.83**	**0.94**	0.29	0.02	0.98
		Alveolar	**0.80**	**0.96**	0.31	0.02	0.99	**0.80**	**0.96**	0.31	0.02	0.99
	**Running 8 km/h**	Total	**0.80**	**0.96**	0.31	0.02	0.99	**0.81**	**0.95**	0.30	0.02	0.99
		Head	**0.81**	**0.95**	0.30	0.02	0.99	**0.82**	**0.95**	0.30	0.02	0.98
		Tracheobronchial	**0.82**	**0.94**	0.30	0.02	0.98	**0.83**	**0.94**	0.29	0.02	0.98
		Alveolar	**0.80**	**0.96**	0.31	0.02	0.99	**0.80**	**0.96**	0.31	0.02	0.99
	**Walking 4 km/h**	Total	**0.80**	**0.96**	0.31	0.02	0.99	**0.81**	**0.95**	0.30	0.02	0.99
		Head	**0.81**	**0.95**	0.30	0.02	0.99	**0.82**	**0.95**	0.30	0.02	0.98
		Tracheobronchial	**0.82**	**0.94**	0.30	0.02	0.98	**0.83**	**0.94**	0.29	0.02	0.98
		Alveolar	**0.80**	**0.96**	0.31	0.02	0.99	**0.80**	**0.96**	0.31	0.02	0.99
	**Standing**	Total	**0.80**	**0.96**	0.31	0.02	0.99	**0.80**	**0.96**	0.31	0.02	0.99
		Head	**0.83**	**0.94**	0.29	0.02	0.98	**0.83**	**0.94**	0.29	0.02	0.98
		Tracheobronchial	**0.83**	**0.94**	0.29	0.02	0.98	**0.82**	**0.94**	0.29	0.02	0.98
		Alveolar	**0.78**	**0.96**	0.32	0.02	0.99	**0.78**	**0.97**	0.32	0.02	0.99
	**Sitting**	Total	**0.80**	**0.96**	0.31	0.02	0.99	**0.80**	**0.96**	0.31	0.02	0.99
		Head	**0.83**	**0.94**	0.29	0.02	0.98	**0.83**	**0.94**	0.29	0.02	0.98
		Tracheobronchial	**0.83**	**0.94**	0.29	0.02	0.98	**0.82**	**0.94**	0.29	0.02	0.98
		Alveolar	**0.78**	**0.96**	0.32	0.02	0.99	**0.78**	**0.97**	0.32	0.02	0.99
***r***	**Yard Work**	Total	**0.90**	**0.98**	0.55	0.15	0.99	**0.90**	**0.98**	0.55	0.15	0.99
		Head	**0.90**	**0.97**	0.55	0.15	0.99	**0.91**	**0.97**	0.55	0.15	0.99
		Tracheobronchial	**0.91**	**0.97**	0.54	0.16	0.99	**0.91**	**0.97**	0.54	0.16	0.99
		Alveolar	**0.89**	**0.98**	0.56	0.15	0.99	**0.89**	**0.98**	0.55	0.15	0.99
	**Running 8 km/h**	Total	**0.90**	**0.98**	0.55	0.15	0.99	**0.90**	**0.98**	0.55	0.15	0.99
		Head	**0.90**	**0.97**	0.55	0.15	0.99	**0.91**	**0.97**	0.55	0.15	0.99
		Tracheobronchial	**0.91**	**0.97**	0.54	0.16	0.99	**0.91**	**0.97**	0.54	0.16	0.99
		Alveolar	**0.89**	**0.98**	0.56	0.15	0.99	**0.89**	**0.98**	0.55	0.15	0.99
	**Walking 4 km/h**	Total	**0.90**	**0.98**	0.55	0.15	0.99	**0.90**	**0.98**	0.55	0.15	0.99
		Head	**0.90**	**0.97**	0.55	0.15	0.99	**0.91**	**0.97**	0.55	0.15	0.99
		Tracheobronchial	**0.91**	**0.97**	0.54	0.16	0.99	**0.91**	**0.97**	0.54	0.16	0.99
		Alveolar	**0.89**	**0.98**	0.56	0.15	0.99	**0.89**	**0.98**	0.55	0.15	0.99
	**Standing**	Total	**0.89**	**0.98**	0.56	0.15	0.99	**0.89**	**0.98**	0.56	0.15	0.99
		Head	**0.91**	**0.97**	0.54	0.16	0.99	**0.91**	**0.97**	0.54	0.16	0.99
		Tracheobronchial	**0.91**	**0.97**	0.54	0.16	0.99	**0.91**	**0.97**	0.54	0.16	0.99
		Alveolar	**0.88**	**0.98**	0.57	0.15	1.00	**0.88**	**0.98**	0.57	0.15	1.00
	**Sitting**	Total	**0.89**	**0.98**	0.56	0.15	0.99	**0.89**	**0.98**	0.56	0.15	0.99
		Head	**0.91**	**0.97**	0.54	0.16	0.99	**0.91**	**0.97**	0.54	0.16	0.99
		Tracheobronchial	**0.91**	**0.97**	0.54	0.16	0.99	**0.91**	**0.97**	0.54	0.16	0.99
		Alveolar	**0.88**	**0.98**	0.57	0.15	1.00	**0.88**	**0.98**	0.57	0.15	1.00

**Table 2:** Coefficient of determination (*R^2^*) and correlation coefficient (*r*) calculated for the regional inhaled deposited dose (PM_2.5_) with respect to the size/fractionated particle number concentrations: nucleation mode (NUCL, 10–25 nm), Aitken mode (AITK, 25–100 nm), accumulation mode (ACCU, 0.1–1 µm), coarse mode (COAR, 1–10s µm), and the total diameter range (Total, 10 nm–10 µm).

	Activity	Region	Male					Female				
			NUCL	AITK	ACCU	COAR	Total	NUCL	AITK	ACCU	COAR	Total
***R* ^2^**	**Yard Work**	Total	0.09	0.09	0.07	**0.95**	0.10	0.09	0.09	0.06	**0.96**	0.10
		Head	0.07	0.06	0.04	**0.97**	0.07	0.07	0.06	0.04	**0.98**	0.07
		Tracheobronchial	0.08	0.07	0.05	**0.97**	0.08	0.08	0.07	0.05	**0.97**	0.08
		Alveolar	0.10	0.10	0.08	**0.94**	0.11	0.09	0.10	0.07	**0.95**	0.11
	**Running 8 km/h**	Total	0.09	0.09	0.07	**0.95**	0.10	0.09	0.09	0.06	**0.96**	0.10
		Head	0.07	0.06	0.04	**0.97**	0.07	0.07	0.06	0.04	**0.98**	0.07
		Tracheobronchial	0.08	0.07	0.05	**0.97**	0.08	0.08	0.07	0.05	**0.97**	0.08
		Alveolar	0.10	0.10	0.08	**0.94**	0.11	0.09	0.10	0.07	**0.95**	0.11
	**Walking 4 km/h**	Total	0.09	0.09	0.07	**0.95**	0.10	0.09	0.09	0.06	**0.96**	0.10
		Head	0.07	0.06	0.04	**0.97**	0.07	0.07	0.06	0.04	**0.98**	0.07
		Tracheobronchial	0.08	0.07	0.05	**0.97**	0.08	0.08	0.07	0.05	**0.97**	0.08
		Alveolar	0.10	0.10	0.08	**0.94**	0.11	0.09	0.10	0.07	**0.95**	0.11
	**Standing**	Total	0.07	0.06	0.04	**0.98**	0.07	0.07	0.06	0.04	**0.98**	0.07
		Head	0.05	0.02	0.01	**1.00**	0.03	0.05	0.02	0.01	**1.00**	0.03
		Tracheobronchial	0.14	0.19	0.17	**0.86**	0.20	0.15	0.21	0.20	**0.83**	0.23
		Alveolar	0.09	0.10	0.08	**0.94**	0.11	0.10	0.10	0.08	**0.94**	0.11
	**Sitting**	Total	0.07	0.06	0.04	**0.98**	0.07	0.07	0.06	0.04	**0.98**	0.07
		Head	0.05	0.02	0.01	**1.00**	0.03	0.05	0.02	0.01	**1.00**	0.03
		Tracheobronchial	0.14	0.19	0.17	**0.86**	0.20	0.15	0.21	0.20	**0.83**	0.23
		Alveolar	0.09	0.10	0.08	**0.94**	0.11	0.10	0.10	0.08	**0.94**	0.11
***r***	**Yard Work**	Total	0.30	0.30	0.26	**0.98**	0.32	0.30	0.30	0.25	**0.98**	0.31
		Head	0.27	0.25	0.21	**0.99**	0.27	0.26	0.24	0.20	**0.99**	0.26
		Tracheobronchial	0.28	0.26	0.22	**0.99**	0.28	0.28	0.27	0.22	**0.98**	0.28
		Alveolar	0.31	0.32	0.28	**0.97**	0.34	0.31	0.31	0.27	**0.97**	0.33
	**Running 8 km/h**	Total	0.30	0.30	0.26	**0.98**	0.32	0.30	0.30	0.25	**0.98**	0.31
		Head	0.27	0.25	0.21	**0.99**	0.27	0.26	0.24	0.20	**0.99**	0.26
		Tracheobronchial	0.28	0.26	0.22	**0.99**	0.28	0.28	0.27	0.22	**0.98**	0.28
		Alveolar	0.31	0.32	0.28	**0.97**	0.34	0.31	0.31	0.27	**0.97**	0.33
	**Walking 4 km/h**	Total	0.30	0.30	0.26	**0.98**	0.32	0.30	0.30	0.25	**0.98**	0.31
		Head	0.27	0.25	0.21	**0.99**	0.27	0.26	0.24	0.20	**0.99**	0.26
		Tracheobronchial	0.28	0.26	0.22	**0.99**	0.28	0.28	0.27	0.22	**0.98**	0.28
		Alveolar	0.31	0.32	0.28	**0.97**	0.34	0.31	0.31	0.27	**0.97**	0.33
	**Standing**	Total	0.26	0.24	0.20	**0.99**	0.26	0.27	0.25	0.20	**0.99**	0.26
		Head	0.21	0.15	0.10	**1.00**	0.17	0.22	0.16	0.11	**1.00**	0.18
		Tracheobronchial	0.37	0.43	0.41	**0.92**	0.44	0.39	0.46	0.44	**0.91**	0.47
		Alveolar	0.31	0.32	0.28	**0.97**	0.33	0.31	0.32	0.29	**0.97**	0.34
	**Sitting**	Total	0.26	0.24	0.20	**0.99**	0.26	0.27	0.25	0.20	**0.99**	0.26
		Head	0.21	0.15	0.10	**1.00**	0.17	0.22	0.16	0.11	**1.00**	0.18
		Tracheobronchial	0.37	0.43	0.41	**0.92**	0.44	0.39	0.46	0.44	**0.91**	0.47
		Alveolar	0.31	0.32	0.28	**0.97**	0.33	0.31	0.32	0.29	**0.97**	0.34

**Table 3:** Coefficient of determination (*R^2^*) and correlation coefficient (*r*) calculated for the regional inhaled deposited dose (PM_10_) with respect to the size/fractionated particle number concentrations: nucleation mode (NUCL, 10–25 nm), Aitken mode (AITK, 25–100 nm), accumulation mode (ACCU, 0.1–1 µm), coarse mode (COAR, 1–10s µm), and the total diameter range (Total, 10 nm–10 µm).

	Activity	Region	Male					Female				
			NUCL	AITK	ACCU	COAR	Total	NUCL	AITK	ACCU	COAR	Total
***R* ^2^**	**Yard Work**	Total	0.03	0.01	0.01	**0.87**	0.01	0.03	0.01	0.01	**0.87**	0.01
		Head	0.01	0.00	0.01	**0.79**	0.00	0.02	0.00	0.01	**0.79**	0.00
		Tracheobronchial	0.02	0.01	0.01	**0.86**	0.01	0.02	0.01	0.01	**0.86**	0.01
		Alveolar	0.04	0.02	0.02	**0.95**	0.03	0.04	0.02	0.02	**0.95**	0.03
	**Running 8 km/h**	Total	0.03	0.01	0.01	**0.87**	0.01	0.03	0.01	0.01	**0.87**	0.01
		Head	0.01	0.00	0.01	**0.79**	0.00	0.02	0.00	0.01	**0.79**	0.00
		Tracheobronchial	0.02	0.01	0.01	**0.86**	0.01	0.02	0.01	0.01	**0.86**	0.01
		Alveolar	0.04	0.02	0.02	**0.95**	0.03	0.04	0.02	0.02	**0.95**	0.03
	**Walking 4 km/h**	Total	0.03	0.01	0.01	**0.87**	0.01	0.03	0.01	0.01	**0.87**	0.01
		Head	0.01	0.00	0.01	**0.79**	0.00	0.02	0.00	0.01	**0.79**	0.00
		Tracheobronchial	0.02	0.01	0.01	**0.86**	0.01	0.02	0.01	0.01	**0.86**	0.01
		Alveolar	0.04	0.02	0.02	**0.95**	0.03	0.04	0.02	0.02	**0.95**	0.03
	**Standing**	Total	0.03	0.01	0.01	**0.89**	0.01	0.03	0.01	0.01	**0.89**	0.01
		Head	0.02	0.01	0.01	**0.87**	0.01	0.02	0.01	0.01	**0.86**	0.01
		Tracheobronchial	0.03	0.01	0.02	**0.89**	0.02	0.03	0.02	0.02	**0.89**	0.02
		Alveolar	0.05	0.03	0.02	**0.96**	0.03	0.04	0.03	0.02	**0.95**	0.03
	**Sitting**	Total	0.03	0.01	0.01	**0.89**	0.01	0.03	0.01	0.01	**0.89**	0.01
		Head	0.02	0.01	0.01	**0.87**	0.01	0.02	0.01	0.01	**0.86**	0.01
		Tracheobronchial	0.03	0.01	0.02	**0.89**	0.02	0.03	0.02	0.02	**0.89**	0.02
		Alveolar	0.05	0.03	0.02	**0.96**	0.03	0.04	0.03	0.02	**0.95**	0.03
***r***	**Yard Work**	Total	0.16	0.09	0.10	**0.93**	0.11	0.16	0.09	0.10	**0.93**	0.11
		Head	0.12	0.05	0.08	**0.89**	0.07	0.12	0.05	0.08	**0.89**	0.07
		Tracheobronchial	0.15	0.08	0.09	**0.93**	0.10	0.15	0.08	0.09	**0.93**	0.10
		Alveolar	0.21	0.15	0.13	**0.98**	0.17	0.21	0.15	0.13	**0.98**	0.17
	**Running 8 km/h**	Total	0.16	0.09	0.10	**0.93**	0.11	0.16	0.09	0.10	**0.93**	0.11
		Head	0.12	0.05	0.08	**0.89**	0.07	0.12	0.05	0.08	**0.89**	0.07
		Tracheobronchial	0.15	0.08	0.09	**0.93**	0.10	0.15	0.08	0.09	**0.93**	0.10
		Alveolar	0.21	0.15	0.13	**0.98**	0.17	0.21	0.15	0.13	**0.98**	0.17
	**Walking 4 km/h**	Total	0.16	0.09	0.10	**0.93**	0.11	0.16	0.09	0.10	**0.93**	0.11
		Head	0.12	0.05	0.08	**0.89**	0.07	0.12	0.05	0.08	**0.89**	0.07
		Tracheobronchial	0.15	0.08	0.09	**0.93**	0.10	0.15	0.08	0.09	**0.93**	0.10
		Alveolar	0.21	0.15	0.13	**0.98**	0.17	0.21	0.15	0.13	**0.98**	0.17
	**Standing**	Total	0.16	0.10	0.10	**0.94**	0.12	0.16	0.09	0.10	**0.94**	0.12
		Head	0.15	0.07	0.08	**0.93**	0.10	0.15	0.07	0.08	**0.93**	0.10
		Tracheobronchial	0.18	0.12	0.12	**0.94**	0.14	0.18	0.13	0.13	**0.94**	0.15
		Alveolar	0.21	0.16	0.14	**0.98**	0.18	0.21	0.16	0.14	**0.98**	0.18
	**Sitting**	Total	0.16	0.10	0.10	**0.94**	0.12	0.16	0.09	0.10	**0.94**	0.12
		Head	0.15	0.07	0.08	**0.93**	0.10	0.15	0.07	0.08	**0.93**	0.10
		Tracheobronchial	0.18	0.12	0.12	**0.94**	0.14	0.18	0.13	0.13	**0.94**	0.15
		Alveolar	0.21	0.16	0.14	**0.98**	0.18	0.21	0.16	0.14	**0.98**	0.18

## Data Availability

Data are available upon request.

## Supplementary Materials

The following are available online at https://www.mdpi.com/article/10.3390/ijerph19074303/s1, S1: geographical location, Figure S1: (a) A Map of Jordan showing the geographical location of Amman. (b) A Map of Amman showing the campus of the University of Jordan (shaded area) and (c) a detailed map of the campus of the University of Jordan, showing the sampling location (shaded area) in the middle of the campus; S2. Aerosol measurements, Figure S2: Experimental penetration efficiency through the sampling lines (tubing and diffusion drier); S3. Data handling; S4. Regional Inhaled Deposited Dose Rate, Figure S3. Size-resolved deposition fraction (*DF*) curves for the respiratory tract of adult subjects: (a) male exercising, (b) male at rest, (c) female exercising, and (d) female at rest. Data were adopted from Löndahl et al. [25] and the ICRP and MPPD models [15,21], Table S1. Minute ventilation (volume of air breathed), *V_E_* (m^3^/h), for adult subjects according to Holmes (1994). The last column indicates the deposition fraction curve used for the listed activity (Figure S3); S5. Summary of the aerosol database and particulate matter concentrations, Figure S4. Time series of the (a) submicron particle number concentration, (b) comparison between the condensation particle counter (CPC) and scanning mobility particle sizer (SMPS) + optical particle sizer (OPS) particle number concentrations, and (c,d) the main particle size fraction concentrations of ultrafine particles (*Dp* < 0.1 µm) and accumulation mode particles (*Dp* 0.1–1 µm), Figure S5. (a) Diurnal pattern of the submicron particle number concentration (0.01–1 µm) and (b) date-time spectrum showing the day-to-day and hour-to-hour variation of the number concentration; the color bar scales the number concentration (cm^−3^), Figure S6. Diurnal pattern of the (a) ultrafine particle number concentration (UFP, diameter < 0.1 µm) and (b) accumulation mode particle number concentration (diameter 0.1–1 µm), Figure S7. Date-time spectra showing the day-to-day and hour-to-hour variation of (a) ultrafine particle number concentration (UFP, diameter < 0.1 µm) and (b) accumulation mode particle number concentration (diameter 0.1–1 µm). The color bar scales the number concentration (cm^−3^), Figure S8. (a) Time series of the coarse mode (diameter 1–10 µm) particle number concentration and (b) date-time spectrum showing the day-to-day and hour-to-hour variation of the number concentration; the color bar scales the number concentration (cm^−3^), Figure S9. Mean particle number size distributions during (a,b) cold period (i.e., December–February) and (c,d) warm period (i.e., May–August). The mean distributions are shown for different times of the day on workdays (left panel) and weekend days (right panel), Table S2: Monthly statistics for the main particle size fractions (concentrations in units of cm^−3^).
