# Supplementary material for: Estimation of the Seasonal Inhaled Deposited Dose of Particulate Matter in the Respiratory System of Urban Individuals Living in an Eastern Mediterranean City
Source: Int J Environ Res Public Health. 2022 Apr 3;19(7):4303. doi: 10.3390/ijerph19074303 (PMC8998606; doi:10.3390/ijerph19074303)
Supplement: Supplementary file 1 [file ijerph-19-04303-s001.zip › ijerph-1627161-supplementary.pdf]

Supplementary Martial

Estimation of the Seasonal Inhaled Deposited Dose of Particulate Matter in the Respiratory System of Urban Individuals Living in an Eastern Mediterranean City

Hussein et al.

S1. Geographical location

Jordan is a small country in the Middle East, located at the crossroads of the Levantine and Arabian regions. Syria borders the country on the north, Iraq on the east, and Saudi Arabia on the east and south. Jordan's only sea outlet, the Gulf of Aqaba, lies to the south, while Palestine is to the west. Jordan is similar in size to Austria or Portugal, with a total area of 96,188 square kilometers including the Dead Sea. Western Jordan has a Mediterranean climate with two short transitional seasons, a hot, dry summer, and a mild, wet winter. However, roughly 75% of the country has a desert environment with annual precipitation of less than 200 mm.

Amman is the capital of Jordan and is located in the northern-western part of the country. The current population of Amman is about 4.5 millions (as per year 2021), which is about 42% of the Jordanian total population (about 10.8 millions). The number of males and females in Amman is roughly about 2.4 millions (50.63 %) and 2.1 (49.37 %); respectively. These fractions are still the same throughout the whole population of Jordan.

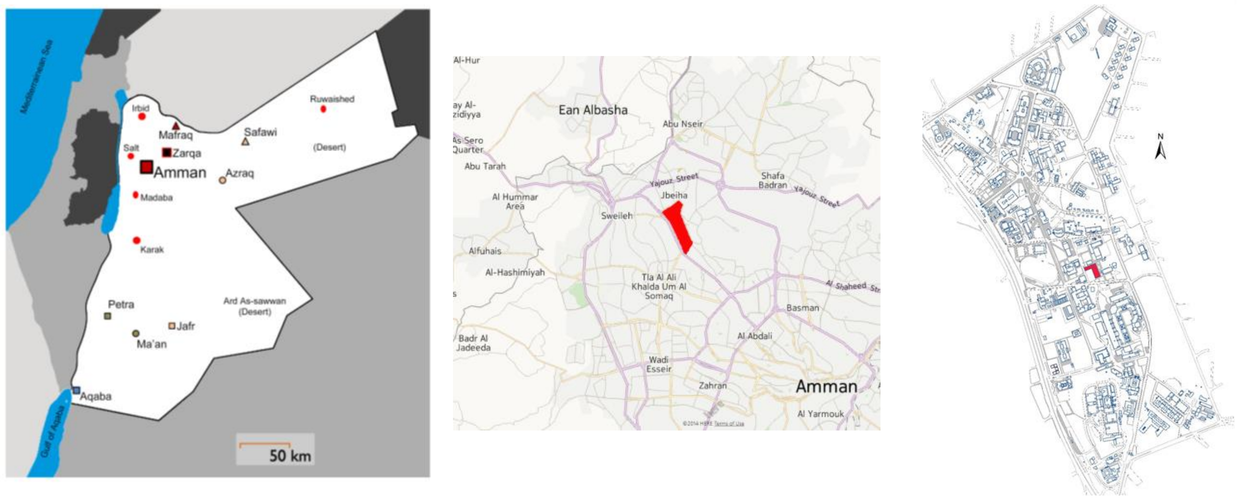

(a) **(b)** **(c)**

Figure S1: (**a**) A Map of Jordan showing the geographical location of Amman. (**b**) A Map of Amman showing the campus of the University of Jordan (shaded area) and (**c**) a detailed map of the campus of the University of Jordan, showing the sampling location (shaded area) in the middle of the campus.

S2. Aerosol measurements

The particle number size distribution was measured with a scanning mobility particle sizer (NanoScan SMPS 3910, TSI, Minnesota, U.S.) and an optical particle sizer (OPS 3330, TSI, Minnesota, U.S.). Using the NanoScan SMPS (electrical equivalent mobility diameter: 0.01–0.42 µm, 13 channels at dry conditions) and the OPS (optical diameter: 0.3–10 µm, 13 channels at dry conditions) can provide a useful setup to monitor a wide particle diameter range 0.01–10 µm. However, combining the measurement results of these two instruments is challenging as will be pointed out in the next section.

The NanoScan SMPS consists of four main built-in components: (1) a cyclone inlet to remove large particles, (2) unipolar particle charger, (3) a radial differential mobility analyzer (RDMA), and (4) an isopropanol-based condensation particle counter (CPC). The particle number size distribution scan was 60 s (45 s upscan and 15 s downscan). The inlet flow rate was 0.75 lpm (±20%) whereas the sample flow rate was 0.25 lpm (±10%).

The OPS measured the particle number size distribution using the TSI default particle size bins, which consisted of 13 equally sized bins based on a lognormal scale. The dead-time correction was applied in the OPS operation. Sampling time-resolution was 5 min with a flow rate ~1 lpm.

The total number concentration of submicron aerosols was measured with a portable condensation particle counter (CPC 3007-2, TSI, Minnesota, U.S.). The cutoff size of this CPC was 10 nm and it was capable of measuring submicron particle number concentration of aerosols with diameters up to 2 µm. According to the specifications provided by the manufacturer, the maximum detectable concentration was 10^5 cm^{-3} with 20% accuracy. The sampling flow rate was 0.1 lpm (inlet flow rate 0.7 lpm).

Each instrument had its own aerosol sampling inlet (~1-m-long and 8 mm inner diameter) which was led through the wall to sample the outdoor aerosols. Each inlet consisted of short Tygon tubes (4 mm inner diameter) connected to a diffusion drier (TSI model 3062-NC). The diffusion drier was used to remove the excess moisture from the aerosol sample.

The aerosol transport efficiency through the aerosol inlet assembly was determined experimentally: ambient aerosol sampling alternatively with and without inlet. The aerosol data was corrected accordingly (Figure S2). The penetration efficiency was ~47% for 10 nm, ~93% for 0.3 µm, and ~40% for 10 µm particles. Accordingly, the particle number size distributions were corrected for losses in the tubing and the diffusion drier.

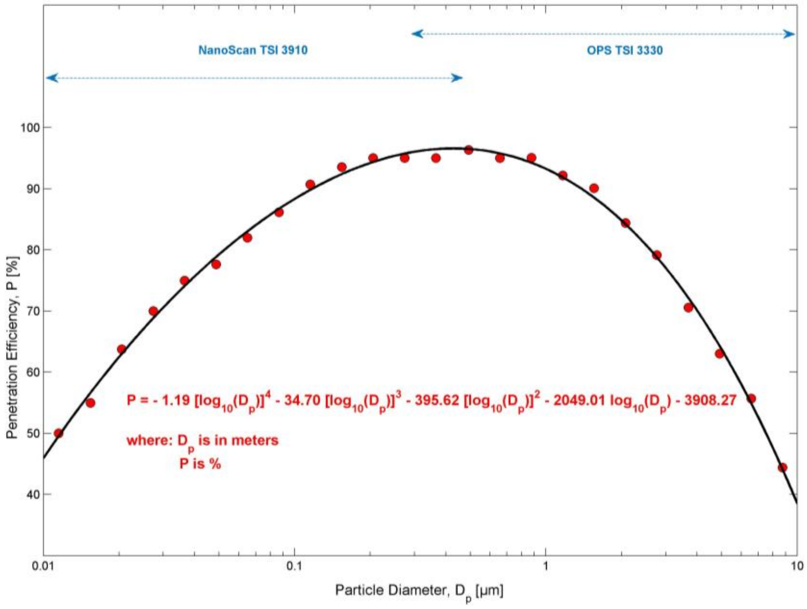

Figure S2: Experimental penetration efficiency through the sampling lines (tubing and diffusion drier).

S3. Data handling

The measurement time-resolution of the SMPS and the CPC was 1 min and of the OPS was 5 min. In order to construct a wide range of the measured particle number size distribution, we performed the following steps: (1) calculated the 5-min average of the SMPS data, (2) omitted the last two channels in the SMPS (i.e., remaining diameter range was 0.01–0.25 μm), (3) omitted the first channel in the OPS (i.e., remaining diameter range was 0.32–10 μm), and (4) merged the two distributions. As such, we obtained a combined particle number size distribution covering the diameter range 0.01–10 μm.

We calculated the particle number concentration (cm^{-3}) within four particle diameter ranges (size-fractionated number concentration): 0.01–0.025 μm (nucleation), 0.025–0.1 μm (Aitken), 0.1–1 μm (accumulation), and 1–10 μm (coarse). Consequently, the total number concentration was obtained as the sum of all these fractions. The size-fractionated number concentrations were obtained by integrating (practically summation) the measured particle number size distribution over the specified particle diameter range

$$PN_{D_{P2}-D_{P1}} = \int_{D_{P1}}^{D_{P2}} n_N^0 d\log_{10}(D_p)\,, \qquad \text{(S1)}$$

where $n_N^0 = dN/d\log_{10}(D_p)$ is the measured particle number size distribution and D_p is the particle diameter.

The processed aerosol data (including all size-fractionated data) was then converted to hourly statistical analysis. This hourly averaged data was then used to calculate the daily and monthly statistical values. The statistical analysis included average, standard deviation, median, minimum, maximum, and percentiles (5%, 25%, 75%, and 95%) of valid number of data points, and percentage of valid data points.

S4. Regional Inhaled Deposited Dose Rate

According to the ICRP and MPPD models, the respiratory tract is divided into three main regions: head/throat, tracheobronchial (TB), and pulmonary/alveolar (P/Alv). Following our previous methods as described by Hussein, et al. (Hussein et al. 2013, Hussein et al. 2015, Hussein et al. 2019, Hussein et al. 2020), we can calculate the regional inhaled deposited dose for a specific particle diameter range (D_{p1}–D_{p2}) during a one-hour exposure period as a dose rate:

$$\text{Dose Rate} = \int_{\mathrm{D_{P}1}}^{\mathrm{D_{P}2}} V_E \times DF(D_P) \times n_N^0(D_P) \times f \cdot \mathrm{dlog}(D_P) \qquad \text{(S2)}$$

where V_E [m^3/h] is the minute ventilation (volume of air breathed, Table S1), $DF(D_p)$ is the particle deposition fraction in a particular region of the respiratory tract (Figure S1), $n_N^0(D_p)$ [particles/cm^3] is the particle number size distribution (i.e., dN/dlog(D_p)), and f is a metric conversion for the particle concentration (i.e., it is 1 for particle number and for particle mass = $\rho_P D_p^3 \pi/6$, where ρ_P is the particle effective density). The deposition fraction (DF) and the particle number size distribution (n) are functions of particle diameter (D_p).

The dose rates were calculated for adult male and female subjects reflecting different types of activities (resting, exercising, and conducting yardwork; Table S1 and Figure S3) and different exposure scenarios.

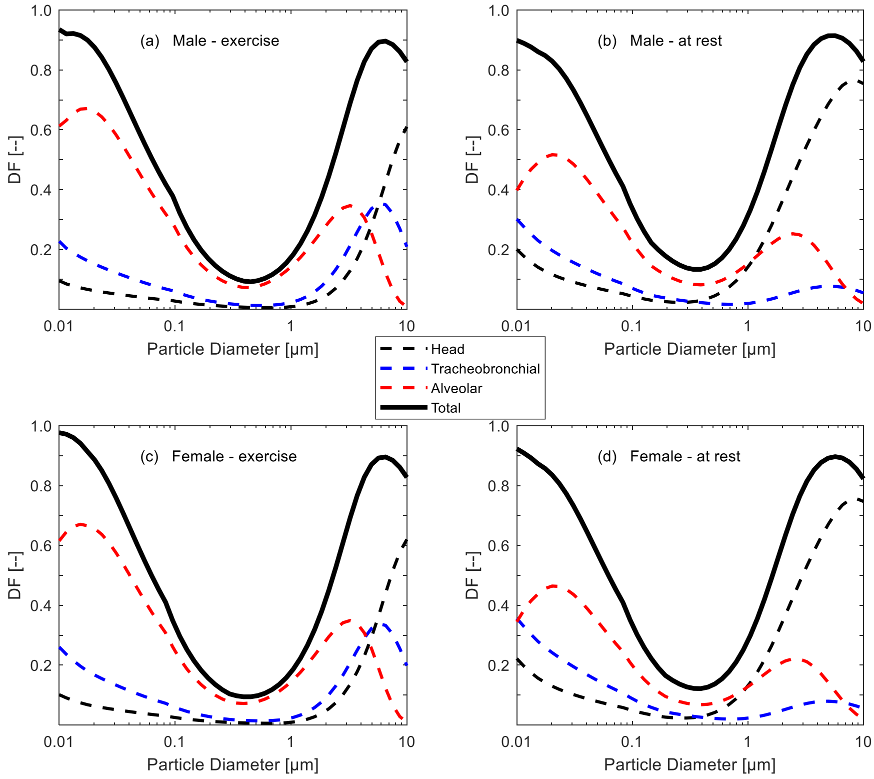

Figure S3. Size-resolved deposition fraction (*DF*) curves for the respiratory tract of adult subjects: (**a**) male exercising, (**b**) male at rest, (**c**) female exercising, and (**d**) female at rest. Data was adopted from Löndahl et al. (2007) and the ICRP and MPPD models (ICRP 1994, Anjilvel and Ashgarian, 1995).

Table S1. Minute ventilation (volume of air breathed), V_E [m^3/h], for adult subjects according to Holmes (1994). The last column indicates the deposition fraction curve used for the listed activity (Figure S3).

Activity	Female	Male	*DF* Curve Type
Yardwork	1.08	1.74	Exercise
Running (8.0 km/h)	3.03	3.48	Exercise
Walking (4.0 km/h)	1.20	1.38	Exercise
Standing	0.48	0.66	at rest
Sitting	0.42	0.54	at rest

S5. Summary about the aerosol data base and particulate matter concentrations

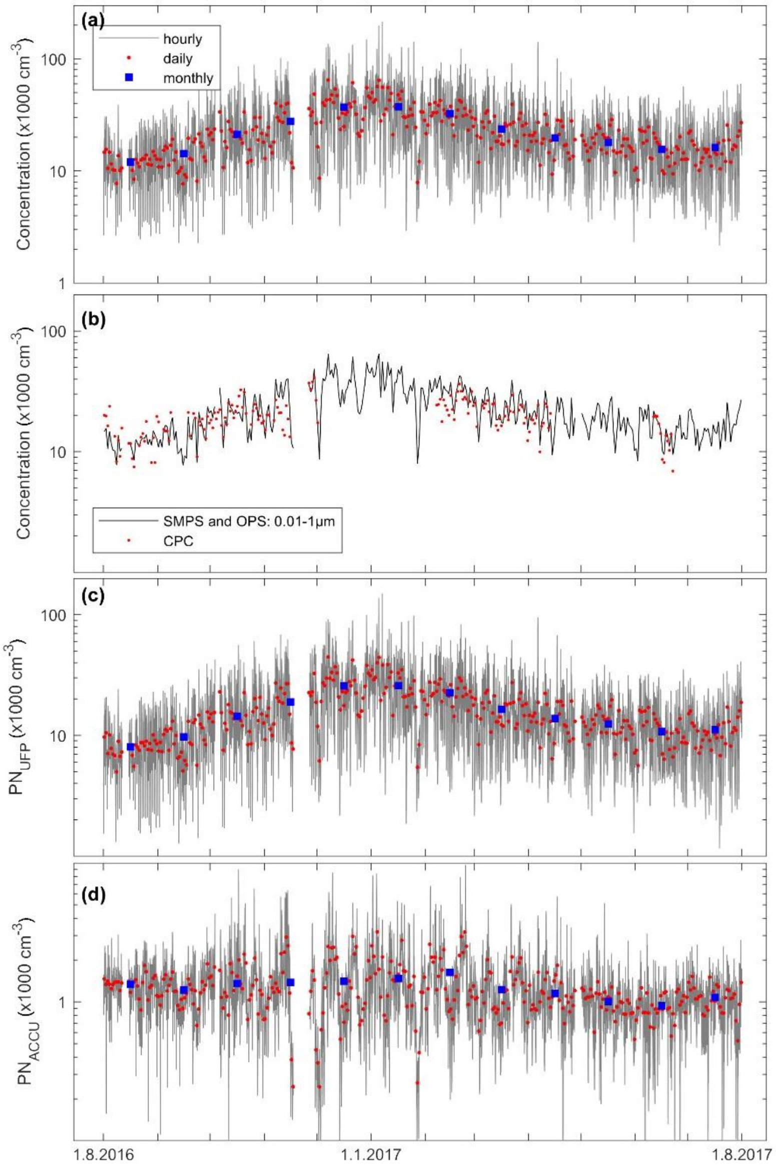

Figure S4. Time series of the (**a**) submicron particle number concentration, (**b**) comparison between the condensation particle counter (CPC) and scanning mobility particle sizer (SMPS) + optical particle sizer (OPS) particle number concentrations, and (**c**,**d**) the main particle size fraction concentrations of ultrafine particles (D_p < 0.1 µm) and accumulation mode particles (D_p 0.1–1 µm).

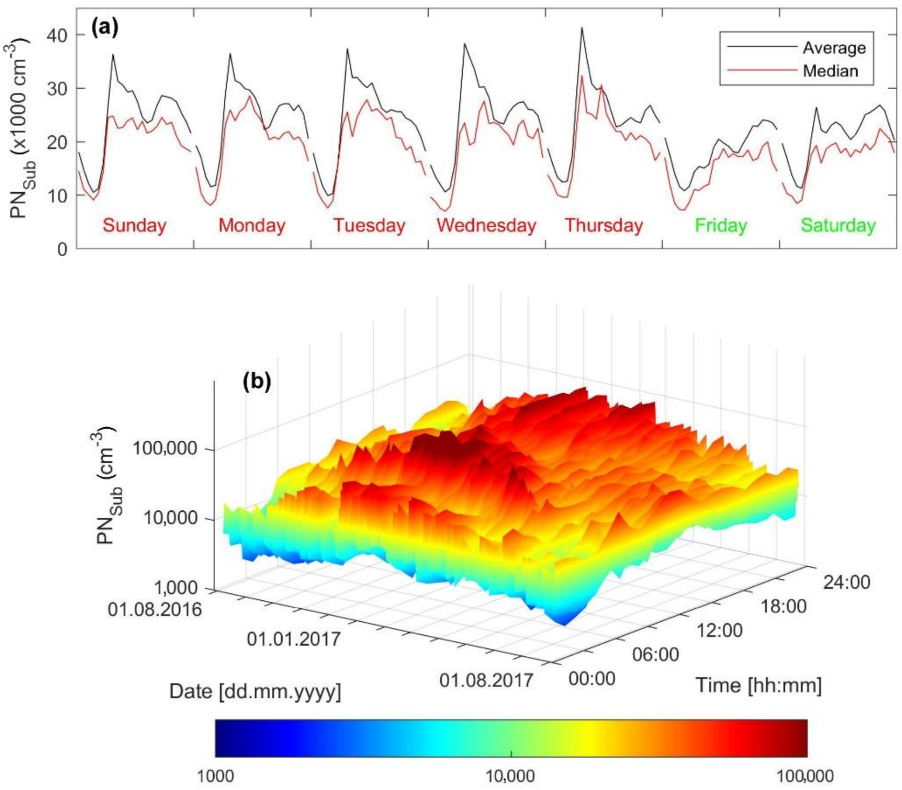

Figure S5. (**a**) Diurnal pattern of the submicron particle number concentration (0.01–1 μm) and (**b**) date-time spectrum showing the day-to-day and hour-to-hour variation of the number concentration; the color bar scales the number concentration (cm^{-3}).

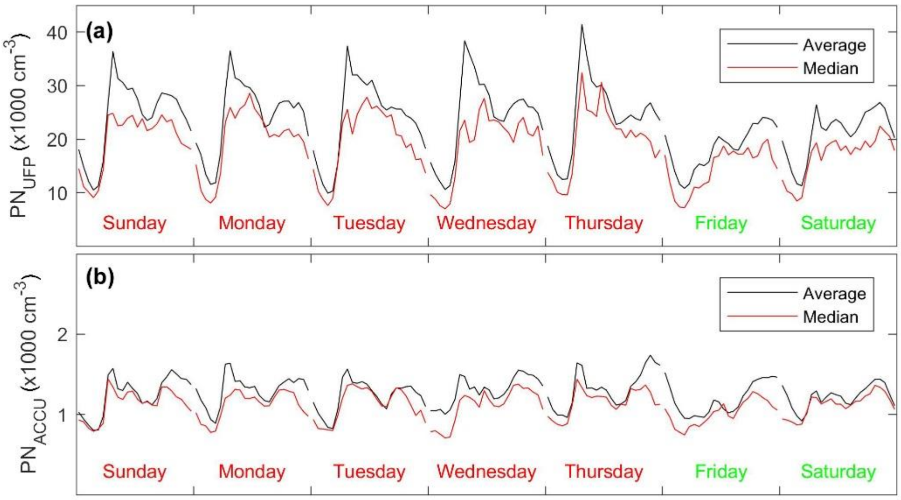

Figure S6. Diurnal pattern of the (**a**) ultrafine particle number concentration (UFP, diameter < 0.1 μm) and (**b**) accumulation mode particle number concentration (diameter 0.1–1 μm).

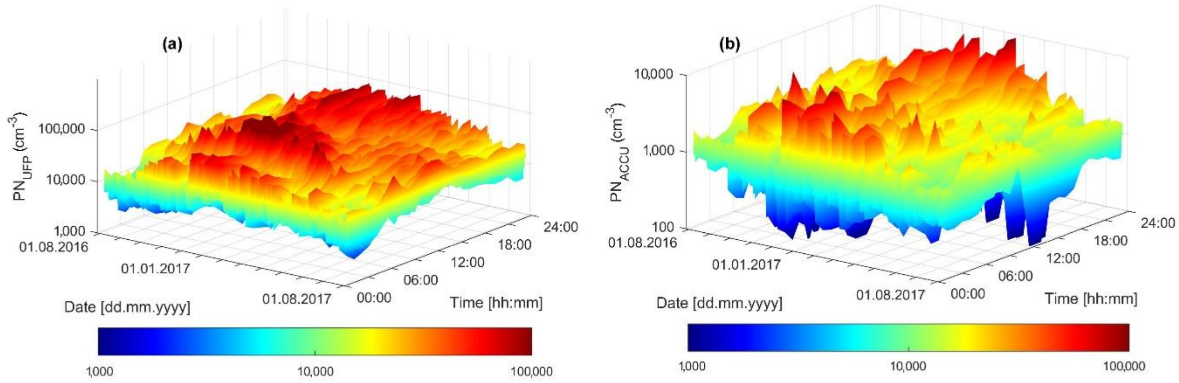

Figure S7. Date-time spectra showing the day-to-day and hour-to-hour variation of (**a**) ultrafine particle number concentration (UFP, diameter < 0.1 μm) and (**b**) accumulation mode particle number concentration (diameter 0.1–1 μm). The color bar scales the number concentration (cm^{-3}).

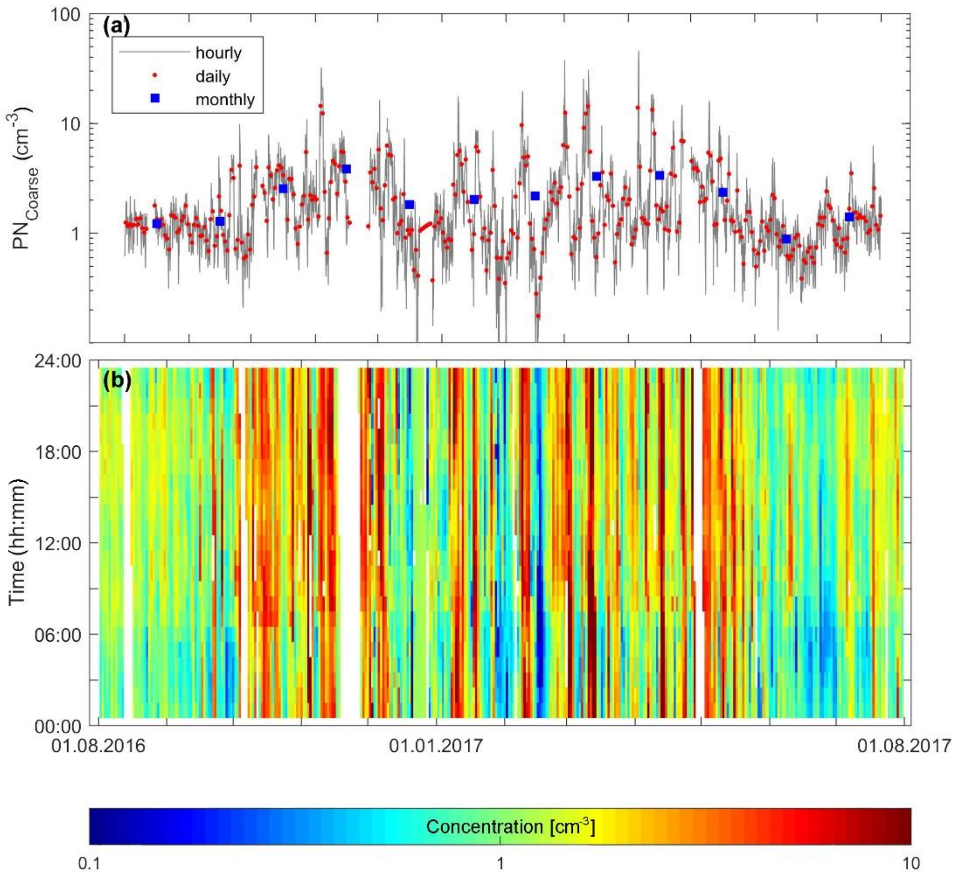

Figure S8. (**a**) Time series of the coarse mode (diameter 1–10 μm) particle number concentration and (**b**) date-time spectrum showing the day-to-day and hour-to-hour variation of the number concentration; the color bar scales the number concentration (cm^{-3}).

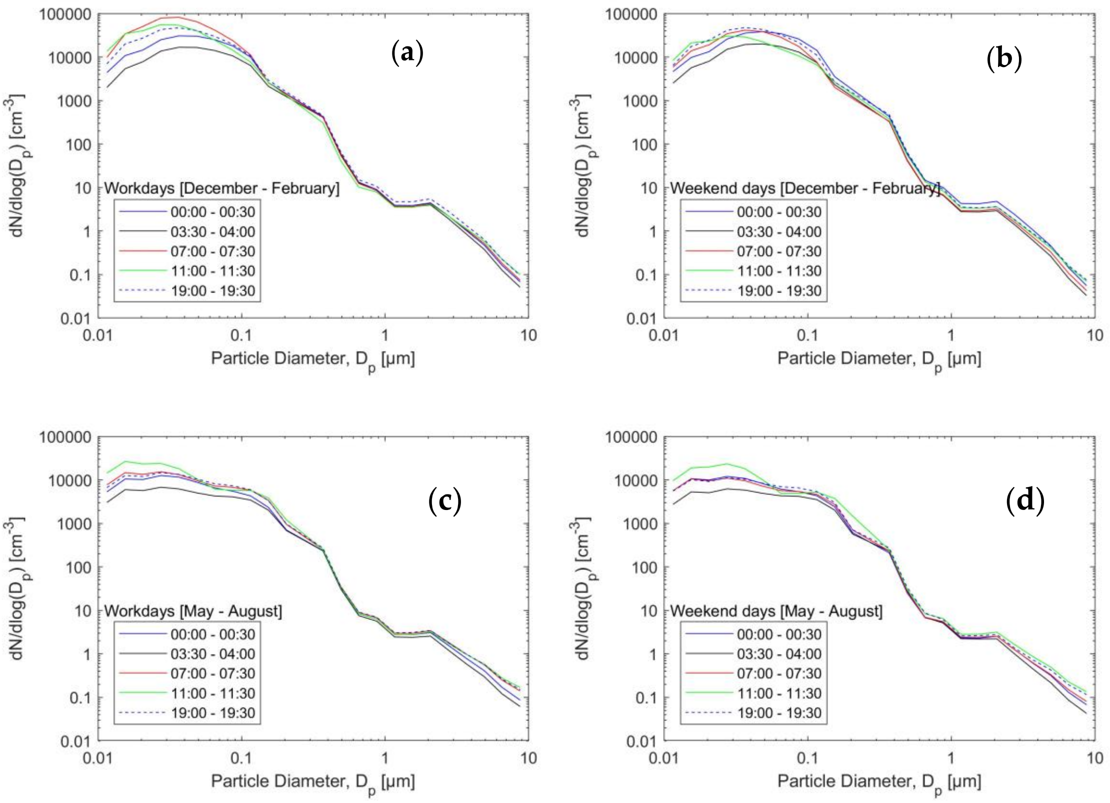

Figure S9. Mean particle number size distributions during (**a**,**b**) cold period (i.e. December–February) and (**c**,**d**) warm period (i.e., May–August). The mean distributions are shown for different times of the day on workdays (left panel) and weekend days (right panel).

Table S2: Monthly statistics for the main particle size fractions [concentrations in units of cm^{-3}].

	Year	Month	Mean	STD	min	5%	25%	Median	75%	95%	Max
Submicron 0.01–1 µm	2016	August	12008	6079	2457	3821	7956	10684	14902	24171	39170
		September	14310	8184	2269	4662	8595	12312	18549	29909	57844
		October	21241	11950	2708	6069	13180	18652	26624	44895	88372
		November	27686	18177	3314	6288	14280	22941	37266	65174	101684
		December	37007	22983	3238	7845	20003	34171	48858	78212	148735
	2017	January	37356	24109	2920	8634	19471	33920	49437	74830	213713
		February	32501	18352	4939	8479	18712	29607	43091	68023	123148
		March	23760	13691	3356	6804	13505	20847	30938	50955	91391
		April	19745	11869	4245	6814	12270	17394	24540	41421	141512
		May	17935	8709	2325	6385	11443	16655	22578	33825	57065
		June	15548	8921	3706	5665	9010	13058	19754	34170	56820
		July	16247	9200	2170	4745	9805	14245	21042	33886	60186
Ultrafine 0.01–0.1 µm	2016	August	8050	4190	1316	2363	5260	7088	10119	16539	24254
		September	9704	5651	1271	3033	5709	8452	12398	20726	39384
		October	14467	7994	1707	4178	9132	12673	18098	29546	60417
		November	18923	12183	2336	4357	9971	15976	25488	43381	68381
		December	25795	15805	2164	5434	14239	23882	33953	55163	99753
	2017	January	25886	16657	1856	5930	13485	23622	34713	51814	149861
		February	22496	12785	3384	5732	13016	20635	29765	47315	83275
		March	16461	9396	2142	4712	9571	14527	21088	35312	61735
		April	13769	8123	2770	4704	8567	12157	17040	28607	95181
		May	12441	5897	1550	4347	8035	11693	15700	23819	38512
		June	10742	6007	2502	3907	6341	9164	13462	23359	35194
		July	11185	6266	1158	3239	6832	9861	14281	23419	41020
Accumulation 0.1–1 µm	2016	August	1337	447	138	653	1044	1326	1594	2134	3056
		September	1216	451	111	606	900	1172	1470	2035	3458
		October	1360	842	102	468	834	1192	1636	2781	9005
		November	1385	1020	141	280	815	1183	1559	3613	6362
		December	1413	1120	87	262	658	1140	1849	3568	8521
	2017	January	1472	1087	80	345	804	1214	1856	3421	8432
		February	1632	1158	198	536	938	1329	1958	3647	9687
		March	1226	691	217	435	752	1104	1538	2355	5052
		April	1148	614	26	497	786	1034	1350	2176	5745
		May	1009	442	184	449	728	940	1212	1781	5137
		June	937	368	28	351	718	920	1118	1572	3112
		July	1076	409	23	456	793	1067	1322	1752	2793
Coarse 1–10 µm	2016	August	1.2	0.4	0.3	0.7	1.0	1.2	1.4	1.9	2.3
		September	1.3	1.2	0.2	0.5	0.7	1.0	1.3	3.8	9.9
		October	2.5	1.4	0.5	0.7	1.5	2.4	3.3	5.1	10.9
		November	3.8	4.1	0.3	0.7	1.5	2.9	4.5	10.8	32.4
		December	1.8	2.0	0.0	0.3	0.9	1.2	1.8	5.9	16.3
	2017	January	2.0	2.3	0.1	0.3	0.7	1.2	2.4	6.6	17.6
		February	2.2	2.5	0.1	0.2	0.7	1.4	2.9	6.4	19.6
		March	3.3	4.7	0.3	0.5	1.0	1.7	3.1	13.9	37.9
		April	3.3	5.1	0.3	0.6	1.1	1.6	3.2	12.0	45.8
		May	2.4	1.9	0.2	0.5	1.1	1.8	3.3	5.4	16.1
		June	0.9	0.6	0.1	0.3	0.6	0.8	1.0	1.8	4.9
		July	1.4	0.7	0.3	0.6	0.9	1.3	1.6	3.0	6.3
